# Porcine reproductive and respiratory syndrome virus nsp5 inhibits the activation of the Nrf2/HO-1 pathway by targeting p62 to antagonize its antiviral activity

**DOI:** 10.1128/jvi.01585-24

**Published:** 2025-02-28

**Authors:** Fang Wang, Fructueux Modeste Amona, Yipeng Pang, Qiaoya Zhang, Yuan Liang, Xiaohan Chen, Yongding Ke, Junhao Chen, Chengchuang Song, Yanhong Wang, Zongyun Li, Chunlei Zhang, Xingtang Fang, Xi Chen

**Affiliations:** 1 Institute of Cellular and Molecular Biology, School of Life Science, Jiangsu Normal University 12675 , Xuzhou, China; 2 College of Veterinary Medicine, Qingdao Agricultural University 98431 , Qingdao, China; University of Michigan Medical School, Ann Arbor, Michigan, USA

**Keywords:** PRRSV, nsp5, Nrf2/HO-1, oxidative stress, p62, Keap1

## Abstract

Porcine reproductive and respiratory syndrome virus (PRRSV) infections often trigger oxidative stress and cytokine storms, resulting in significant tissue damage that causes fatalities in piglets and reproductive issues in sows. However, it is still unknown how oxidative stress is regulated by viral and host factors in response to PRRSV infection. Here, we found that PRRSV induced cellular oxidative stress by triggering the production of reactive oxygen species and inhibiting the expression of antioxidant enzymes. Although Nrf2 is an important redox regulator that initiates the expression of downstream antioxidant genes, PRRSV can impair the Nrf2/HO-1 pathway. The overexpression of Nrf2 showed a significant anti-PRRSV effect, and inhibiting the expression of Nrf2 promoted the proliferation of PRRSV. Further analysis showed that Nrf2 positively regulated the production of type I interferons and interferon-stimulated genes, which may contribute to its anti-PRRSV effect. By screening the PRRSV-encoded protein, we found that the PRRSV nsp5 protein can degrade Nrf2 at the protein level. Mechanistically, nsp5 promotes Nrf2-Keap1 binding affinity by inhibiting p62-mediated Keap1 sequestration and increasing Keap1 expression. Subsequently, this increased Keap1-mediated degradation of Nrf2 ubiquitination through K48-linked polyubiquitin. Furthermore, we found that the residues Tyr146 and Arg147 of nsp5 are crucial for inhibiting the activation of the p62-mediated Nrf2 antioxidant pathway. Thus, our findings uncover a novel mechanism by which PRRSV disrupts the host antioxidant defense system and highlight the crucial role of the Nrf2/HO-1 antioxidant pathway in host defense against PRRSV.

**IMPORTANCE:**

Oxidative stress-induced redox imbalance is a crucial pathogenic mechanism in viral infections. Nrf2 and its antioxidant genes serve as the main defense pathways against oxidative stress. However, the role of Nrf2 in the context of porcine reproductive and respiratory syndrome virus (PRRSV) infection remains unclear. In this study, we demonstrated that PRRSV infection decreased the expression of antioxidant genes of the Nrf2 signaling pathway and overexpression of Nrf2 triggered a strong anti-PRRSV effect. PRRSV nsp5 enhanced Keap1-dependent degradation of Nrf2 ubiquitination, thereby weakening cellular resistance to oxidative stress and antagonizing the antiviral activity of Nrf2. Our study further revealed a new mechanism by which PRRSV evades host antiviral innate immunity by disturbing cellular redox homeostasis, providing a new target for developing anti-PRRSV drugs.

## INTRODUCTION

Porcine reproductive and respiratory syndrome (PRRS) is caused by an enveloped, single-stranded, positive-sense RNA virus, classified under the *Nidovirales* order, family *Arteriviridae*, and genus *Betaarterivirus* (1, 2). The porcine reproductive and respiratory syndrome virus (PRRSV) has a genome size of approximately 15.4 kb (2) and 12 known open-reading frames (ORFs) encoding 8 structural proteins and 17 non-structural proteins (nsps) (3–5). These nsps are crucial for viral replication, virulence, host immunosuppression, and genomic transcription regulation (6). Among these nsps, nsp5, a hydrophobic transmembrane protein, plays a crucial role in PRRSV infection. It forms a membranous structure in the cytoplasm, potentially facilitating PRRSV replication (7). Nsp5, although an incompletely understood membrane protein, has been found to trigger transfected cell death through autophagy and is linked to autophagy induced by viruses (8, 9). PRRSV nsp5 has also been reported to antagonize STAT3 signaling by hastening its degradation via the ubiquitin (Ub)-proteasome pathway, with the C-terminal domain of nsp5 being essential for this process (10). Further investigation is needed to explore the function of nsp5 in other pathways and understand its role in PRRSV infection and its potential as a target for therapeutic intervention.

PRRSV infection causes fatalities in growing pigs, particularly piglets, due to acute respiratory failure and reproductive failures in sows (11, 12). Viral respiratory infections often increase reactive oxygen and nitrogen species (ROS and RNS), leading to oxidative stress (13, 14) and cytokine storms (15). Subsequently, heightened oxidative stress decreases the host antiviral response, increases viral replication, and causes virus-induced cell and tissue injury apoptosis and inflammation, leading to organ damage (13, 16). Oxidative stress arises from an imbalance between oxidant production and antioxidant defense systems, leading to metabolic and physiological changes and irreversible cell damage (17, 18). Increasing evidence suggests that the host cell’s oxidative stress status significantly influences viral replication and infectivity (19–21). Studies have shown that oxidative stress exacerbates and prolongs the cytokine storm in severe coronavirus disease 2019 (COVID-19) patients (22, 23). PRRSV infection has been shown to induce oxidative stress in cells by generating ROS, which activates the NF-κB pathway, a crucial aspect of PRRSV pathogenesis (24, 25). In addition, previous studies have found that dengue virus infection downregulates endogenous antioxidant enzyme activities, leading to the collapse of redox homeostasis and the development of infection to more severe forms of disease (26, 27). However, the exact mechanism behind the oxidative stress induced by PRRSV infection remains unclear.

Nuclear factor (erythroid-derived 2)-like 2 (Nrf2) is a transcription factor that controls redox balance in animal cells, and it’s tightly regulated by Kelch-like ECH-associated protein-1 (Keap1) (28). The Keap1-Nrf2 pathway is an important cell defense against oxidative and electrophilic stressors (29). In response to oxidative stress, Keap1 becomes inactive, releasing Nrf2 to induce Nrf2-responsive genes. Typically, these genes shield against stress-induced cell death, positioning Nrf2 as the primary regulator of tissue damage during infection (30, 31). Keap1-Nrf2 interaction can be disrupted by oxidants, allowing Nrf2 translocation to the nucleus, enhancing the expression of antioxidant response elements (ARE) crucial for detoxification reactions, cell survival, and immune modulation (32). Furthermore, various processes, some Keap1 independent, contribute to Nrf2 activation or inactivation (28). The autophagic degradation of p62/SQSTM1 can modulate Keap1-Nrf2, forming the p62-Keap1-Nrf2 axis (33, 34). Nrf2-mediated ARE responses have been observed to impact the outcome of various viral infections (35, 36). However, the mechanisms through which Keap1-Nrf2-related signals protect against PRRSV infection and how PRRSV influences the antioxidant response remain incompletely understood.

In this study, we demonstrated that PRRSV nsp5 impairs the activation of the Nrf2/HO-1 pathway by targeting p62. PRRSV infection induced oxidative stress in cells, leading to increased levels of ROS and MDA and decreased levels of GSH and SOD. Furthermore, PRRSV infection inhibits the activation of the Nrf2/HO-1 axis, which is essential for inducing antiviral responses by regulating the production of interferon-β (IFN-β) and interferon-stimulated genes (ISGs). PRRSV nsp5 impedes p62-mediated sequestration of Keap1, promoting the degradation of Nrf2 ubiquitination, which in turn antagonizes the antiviral activity of Nrf2. The Y146 and R147 residues of nsp5 play an important role in inhibiting the p62-mediated Nrf2 antioxidant pathway. Altogether, these findings uncover a novel inhibitory function of PRRSV nsp5 on Nrf2-mediated signaling and may provide clues for the development of new strategies against PRRSV infection.

## MATERIALS AND METHODS

### Cells and viruses

PAMs (primary alveolar macrophages) were isolated from lung lavage fluid of 4- to 6-week-old healthy euthanized piglets, as they are the main target cells for PRRSV *in vivo* as previously described (36). The PAMs were cultured in RPMI 1640 medium (Gibco, New York) supplemented with 10% heat-inactivated fetal bovine serum (FBS, Gibco), 100 U/mL penicillin, and 100 mg/mL streptomycin (Biosharp). The African green monkey kidney cell line Marc-145 (China Center for Type Culture Collection, China) and HEK-293T cells were cultured in Dulbecco’s modified Eagle medium (DMEM; SenBeiJia) supplemented with 10% FBS, penicillin, and streptomycin as mentioned earlier. All cells were maintained at 37°C in a humidified atmosphere with 5% CO_2_.

Three North American genotype 2 PRRSV strains were utilized in this research. All experiments were carried out using the highly pathogenic PRRSV strain BB0907 (GenBank accession number HQ315835.1), referred to as “PRRSV” in this study. Additionally, the PRRSV strains S1 (GenBank accession number DQ459471.1) and FJ1402 (GenBank accession number KX169191.1) were also employed. The PRRSV strains were propagated in Marc-145 cells and titrated using the 50% tissue culture infective dose (TCID_50_) method (37).

### Pigs were challenged with the PRRSV BB0907 strain

Eighteen 4-week-old PRRSV-negative pigs were randomly divided into two groups: the PRRSV-infected group (*n* = 12) and the mock-infected control group (*n* = 6). Each group of pigs was kept in an isolated room with appropriate temperature, humidity, and independent ventilation and was free to drink and eat. After 1 week of adaptive feeding, nasal cannula instillation (2 mL:1 × 10^5^ TCID_50_ virus /mL) was used. Pigs in the mock-infected control group were inoculated with DMEM, the supernatant of Marc-145 cells, at the same dose in the same way. At 7, 14, and 21 days after infection (dpi), we selected two and three pigs from the control group and PRRSV-infected group, respectively, and euthanized them humanely with excessive pentobarbital. Lung tissue was used for hematoxylin and eosin (H&E) staining and immunohistochemistry (IHC).

### Plasmid construction and transfection

The Nrf2 gene was extracted from PAMs cDNA and subcloned into the pCAGGS vector. The p62 was also derived from PAMs cDNA and subcloned into the pCAGGS-HA vector, adding an N-terminal HA epitope tag. Similarly, the Keap1 was extracted from PAMs cDNA and subcloned into the pCAGGS-Myc vector, providing an N-terminal Myc epitope tag. The pCAGGS-Flag vector was used to clone genes encoding PRRSV BB0907 nsp1–nsp5, nsp7, nsp9–nsp12, GP2, GP3, GP4, GP5, M, and N. Four nsp5 fragments (D1: residues 1 to 72, D2: residues 59 to 170, D3: residues 1 to 122, and D4: residues 86 to 170) were subcloned into the pCAGGS-Flag vector. Prime STAR GXL DNA Polymerase (TaKaRa, R050A) was utilized to generate alanine substitution mutations within the nsp5. All constructs were confirmed through DNA sequencing, and the primers employed for plasmid construction are listed in Table S1. The HA-Ub, HA-Ub_K48_, HA-Ub_K48R_, and HA-Ub_K63_ mutant plasmids were purchased from MiaoLing Biology. These plasmids were transiently transfected into cells using Lipofectamine 8000 (Beyotime, C0533) according to the manufacturer’s protocol. Marc-145 and HEK-293T cells were seeded in six-well plates and grown to 60%–70% confluence.

### RNA interference assay

Small interfering RNAs targeting p62 and Keap1, as well as scrambled siRNA (siNC), were originated and produced by BioSune (Shanghai). These siRNAs were transfected at a final concentration of 10 nM into 2 × 10^6^/Marc-145 cells and HEK-293T adherent cells per well using Lipofectamine RNAiMAX (Invitrogen) according to the manufacturer’s instructions. The siRNA sequences are listed in Table S2.

### Real-time quantitative PCR

Total RNA was extracted from cells at the indicated time points post-infection using TRIzol reagent (Tiangen, China). One microgram of total RNA was reverse transcribed into cDNA using an Evo M-MLV reverse transcription kit (Accuratebio, AG11728) according to the manufacturer’s instructions. Real-time quantitative PCR (RT-qPCR) was performed using a QuantStudio3 system (Applied Biosystems) and SYBR Green qPCR (Accuratebio, AG11718). Data were normalized to GAPDH mRNA levels in each sample and performed in triplicate. Relative mRNA expression was calculated using the 2^−ΔΔCt^ method. Reverse transcription primers are listed in Table S3.

### ROS measurement assay

ROS levels were measured using a Reactive Oxygen Species Assay Kit (Beyotime, S0033S) according to the manufacturer’s instructions. Marc-145 cells were infected with PRRSV (multiplicity of infection [MOI] of 1) for 48 h and then incubated with 10 µM DCFH-DA at 37°C for 30 min. The cells were washed and imaged using an inverted fluorescence microscope (U-RFL-T, Olympus, Japan).

### MDA, GSH, and SOD assays

SOD, GSH, and MDA levels in cells were measured using the SOD Assay Kit (A001-3-2), GSH Assay Kit (A006-2-1), and MDA Assay Kit (A003-4-1), respectively, according to the manufacturer’s instructions (Nanjing Jiancheng, China). These levels were standardized to protein concentrations determined by the bicinchoninic acid Protein Assay Kit (Beyotime, P0010S).

### Western blotting

Cells were harvested after treatment for the indicated time and lysed with cell lysis buffer (Beyotime, P0013B) for 16 min. Equal amounts of protein were separated by 10% SDS-PAGE and then transferred to polyvinylidene difluoride membranes (GVS, 1212639). Membranes were blocked with 5% non-fat milk and incubated overnight at 4°C with specific primary antibodies: anti-PRRSV N antibody (GTX637947); anti-Nrf2 (T55136), anti-Flag-tag (M20008), anti-IRF3 (T55779), anti-p-IRF3 (TA2436), anti-STAT1 (T55227), anti-p-STAT1 (T55702), and anti-p-STAT3 (T56566) purchased from Abmart; anti-β-actin (66009), anti-HO-1 (10701), anti-Keap1 (10503), anti-p62 (18420), anti-p65 (10745), anti-p-p65 (82335), anti-HA-tag (51064), anti-Myc-tag (10828), anti-STAT2 (16674), anti-STAT3 (10253), anti-ISG15 (15981), anti-IFITM1 (60074), anti-IFIT3 (15201), anti-GBP1 (15303), anti-USP18 (12153), and anti-MX1 (13750) purchased from Proteintech; anti-ubiquitin (A19686), anti-ubiquitin-K48 (A3606), and anti-ubiquitin-K63 (A18164). Following primary antibody incubation, membranes were washed three times with TBST buffer. They were then incubated with secondary antibodies, HRP-conjugated goat anti-mouse IgG polyclonal Antibody (HuaAnbio, HA1006), or HRP-conjugated goat anti-rabbit IgG polyclonal Antibody (HuaAnbio, HA1001) for 1 h at room temperature. A system based on enhanced chemiluminescence was used to visualize immunoreactive proteins (Beyotime, P0018M).

### Co-immunoprecipitation assay

Cells were transfected or co-transfected with the plasmids for 36 h and then harvested for co-immunoprecipitation. Cells were washed with cold PBS and lysed in cell lysis buffer (Beyotime, P0013) supplemented with phenylmethylsulfonyl fluoride (Beyotime, ST506). Immunoprecipitation was performed using an immunoprecipitation kit (Protein A + G magnetic beads method) (Beyotime, P2197) according to the manufacturer’s instructions. Specifically, 3 µg of the indicated tag antibodies were added to 20 µL of magnetic beads and incubated at room temperature for 1 h. After removing the supernatant, cell lysates containing the antigen were incubated with the bead-antibody complex overnight at 4°C. The bead-antibody-antigen complex was washed three times, and the remaining complexes were eluted with SDS loading buffer and separated by 10% SDS-PAGE. Western blotting analysis was performed on the precipitates and whole-cell lysates as described above.

### Immunofluorescence assay

Cells were fixed with 4% paraformaldehyde (Biosharp, China) for 10 min and permeabilized with 0.5% Triton X-100 (Solarbio, China) in PBS for 15 min. After washing with PBS, cells were blocked with 5% bovine serum albumin at room temperature for 1 h. After blocking, cells were incubated with the appropriate primary antibodies overnight at 4°C. Following three washes with PBS, cells were incubated with the indicated secondary antibodies at room temperature for 1.5 h. Nuclei were additionally stained with DAPI (49,69-diamidino-2-phenylindole; Beyotime) for 5 min. All images were captured and processed using a confocal laser scanning microscope (SP8, Leica, Germany).

### Luciferase reporter assay

Marc-145 cells were cultured in 24-well plates and grown to 60%–70% confluence. The cells were then co-transfected with 800 ng of Nrf2 expression plasmid or an empty vector, 100 ng of IFN-β luciferase reporter plasmid, and 20 ng of Renilla luciferase plasmid (as an internal control). After 24 h, cells were either mock infected or infected with PRRSV for 10 h or mock stimulated or stimulated with poly(I:C) for 8 h. The cells were subsequently lysed with luciferase lysis buffer, and luciferase activity was measured using a dual-luciferase reporter assay kit (Vazyme, DL101) according to the manufacturer’s instructions. Relative luciferase activity was normalized to Renilla luciferase activity. Each treatment was performed in triplicate.

### Nuclear/cytoplasmic fractionation assay

Marc-145 cells were infected with PRRSV (MOI = 1) and collected at 48 h post-infection (hpi). Nuclear and cytoplasmic protein samples were extracted from the harvested cells using a nuclear and cytoplasmic protein extraction kit (Beyotime, P0027) according to the manufacturer’s instructions. The protein expression of Nrf2 in the nuclear and cytoplasmic fractions was detected by western blot. Histone H3 was used as an internal nuclear control, and β-actin was used as a cytoplasmic internal control.

### RNA-sequencing analysis

PAMs were infected with PRRSV at 1 MOI, and cells were collected for transcriptome analysis 24 hpi. Three biological replicates were performed for each sample. RNA isolation and sequencing were conducted by Guangzhou Genedenovo Biotechnology Co. Ltd., and gene expression data were obtained using the Illumina NovaSeq 6000 sequencer. Bioinformatics analysis was performed using the omicsmart tool available at https://www.omicsmart.com/#/.

### Molecular docking analysis

The structures of nsp5 and p62 were predicted using AlphaFold (https://alphafold.ebi.ac.uk/). The binding mode between nsp5 and p62 was studied using the HDOCK website (http://hdock.phys.hust.edu.cn/). Based on the docking scores provided by the HDOCK server, the best predicted binding mode was selected to analyze the detailed interaction network between these two proteins. The best predicted binding mode was visualized, analyzed, and graphed using the PYMOL program (https://www.schrodinger.com/pymol).

### H&E assay

The lung tissues of piglets were isolated and fixed with 4% formaldehyde for 24 h. They were dehydrated, embedded in paraffin, and sliced into 8 mm thick sections. According to the manufacturer’s instructions, sections were rehydrated and stained with hematoxylin and eosin (Beyotime, C0105s) for observing pathological changes, while other sections proceeded for IHC. The images were acquired using an Olympus microscope.

### IHC assay

The IHC was performed to quantify the Nrf2 signal in lung tissue. This quantification involved measuring the intensity of the staining, which correlates with Nrf2 protein levels. The porcine lung tissue slices were put on silane-coated slides, dewaxed, and rehydrated with ethanol at a decreasing concentration. Antigen retrieval was performed by incubating the section with 10 mM citrate buffer using heat-induced methods as per the manufacturer’s instructions. Nrf2 expression was measured using a primary antibody specific to Nrf2 (Proteintech, 16396).

### Statistical analysis

Data were analyzed using GraphPad Prism 5.0. Statistical significance was calculated using the two-tailed Student’s *t*-test or one-way analysis of variance. Differences were considered statistically significant if the *P* value was less than 0.05.

## RESULTS

### PRRSV induces oxidative stress and inhibits Nrf2/HO-1 axis activation

**Fig 1:**
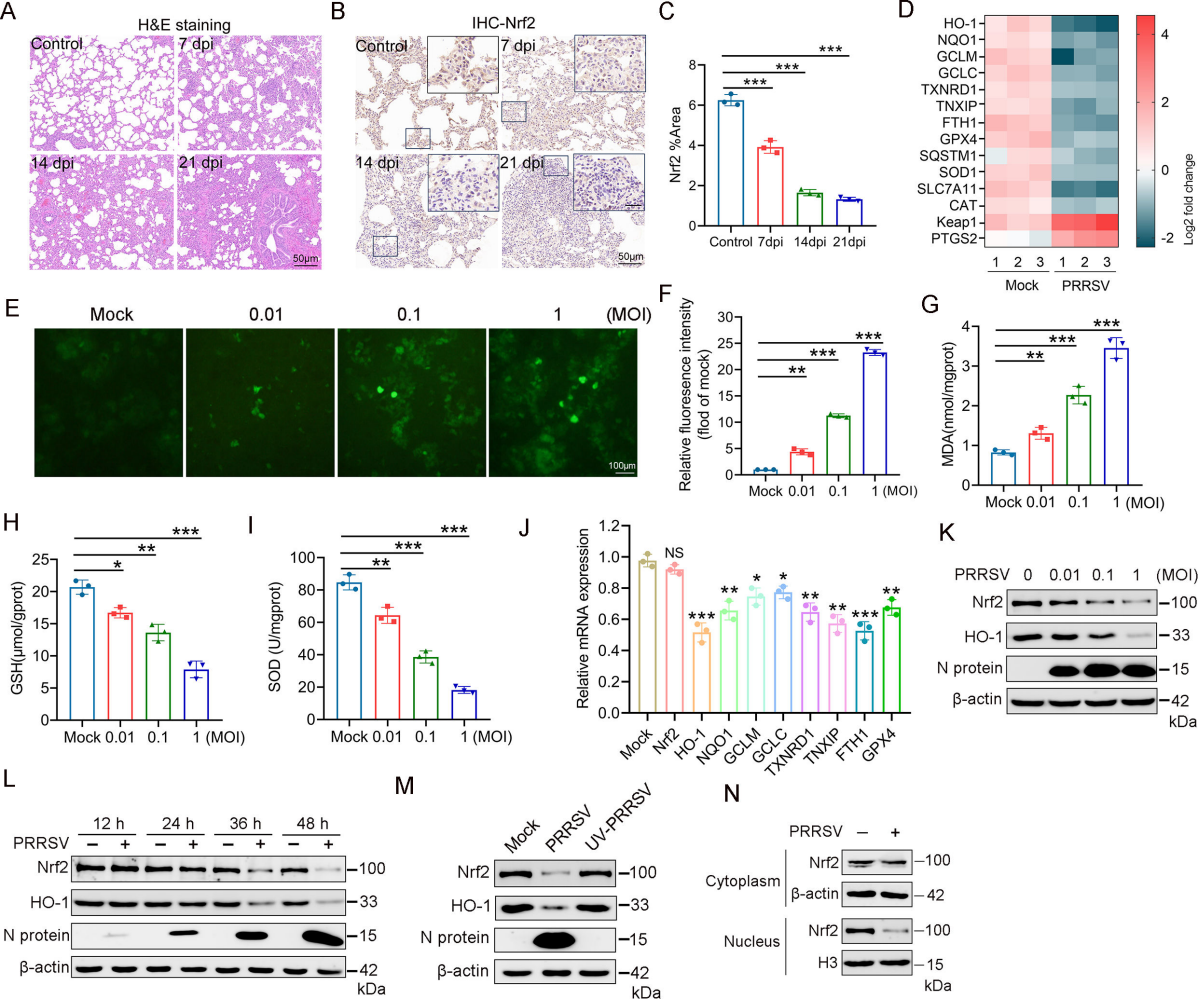
PRRSV infection induces oxidative stress and inhibits Nrf2/HO-1 axis activation. (**A**) Representative micrographs of lung tissue from mock-infected and PRRSV-infected piglets (7, 14, and 21 dpi) showed histopathological changes. Lung tissue was stained with H&E. (**B**) Using an anti-Nrf2 antibody, the Nrf2 antigen in piglet lung tissue was examined by IHC. (**C**) The relative intensity of Nrf2 was quantified using ImageJ software. (**D**) The heatmap shows the Nrf2-driven genes in mock-infected and PRRSV-infected PAMs. Color intensity represents row-scaled normalized log2 (CPM) expression values, and columns show data for each of the three replicates. (**E**) Marc-145 cells were infected with PRRSV (MOI = 1) and at 48 hpi. ROS generation was measured using the fluorescent probe DCFH-DA. Images were captured using an inverted fluorescence microscope (Olympus). (**F**) The relative intensity of fluorescence was quantified using ImageJ software. (**G–I**) Marc-145 cells were infected with PRRSV at MOI of 0.01, 0.1, or 1. At 48 hpi, MDA, SOD, and GSH levels in the cells were measured. (**J**) Effect of PRRSV infection on the mRNA expression of Nrf2 and its downstream antioxidant molecules. (**K and L**) Effect of PRRSV infection on Nrf2 and HO-1 expression levels. PAMs were infected with PRRSV at MOI of 0.01, 0.1, or 1 for 48 h (**K**) or an MOI of 1 for 12, 24, 36, and 48 hpi (**L**). Total proteins were subjected to western blot analysis using antibodies against Nrf2, HO-1, N protein, and β-actin. (**M**) Ultraviolet (UV)-inactivated PRRSV does not affect Nrf2 expression. PRRSV was inactivated by UV light for 2 h, and PAMs were infected with either UV-inactivated or non-inactivated virus. Cells were harvested at 48 hpi for western blot analysis. (**N**) Subcellular fractionation analysis was performed, followed by immunoblot analysis of cytoplasmic and nuclear fractions. Data are expressed as means ± SD of three independent experiments (***P < 0.001, **P < 0.01, and *P < 0.05).

As a transcription factor involved in the regulation of numerous redox and antioxidant proteins, Nrf2 plays a key role in managing oxidative stress. First, a PRRSV challenge was performed to confirm whether PRRSV infection could trigger oxidative stress *in vivo*. Immunohistochemical analysis of lung tissue injury showed that the PRRSV-infected group had more severe inflammatory injury compared with the mock-infected control group, including inflammatory cell infiltration, interstitial pneumonia, congestion, and narrowing of the alveolar space, ultimately leading to the disappearance of lung structure (Fig. 1A). Immunohistochemical experiments using the Nrf2 antibody showed a relatively abundant Nrf2 signal in the control group, while the PRRSV-infected piglet lung showed a suppressed Nrf2 signal (Fig. 1B and C).

Next, we explored the effect of PRRSV infection on cellular oxidative stress *in vitro*. High-throughput RNA sequencing of PAMs post-PRRSV infection revealed that genes related to Nrf2-dependent antioxidant response were suppressed in PAMs (Fig. 1D). Marc-145 cells were infected with PRRSV at MOI of 0.01, 0.1, and 1. At 48 hpi, intracellular ROS levels were measured using the fluorescent probe DCFH-DA. Confocal laser scanning microscopy revealed a dose-dependent increase in cellular fluorescence intensity with increasing infection doses, suggesting elevated ROS (Fig. 1E and F). Additionally, MDA, a biomarker of lipid peroxidation and oxidative stress, exhibited a dose-dependent increase following PRRSV infection (Fig. 1G). PRRSV infection also led to a reduction in the antioxidant enzymes GSH and SOD (Fig. 1H and I). These data collectively suggest that PRRSV infection induces oxidative stress.

To investigate the impact of PRRSV infection on Nrf2 expression, PAMs were infected with PRRSV at 1 MOI for 36 h. RT-qPCR analysis revealed that PRRSV infection had no significant effect on Nrf2 mRNA expression levels. However, it significantly suppressed the mRNA expression of Nrf2-regulated downstream antioxidant molecules, including HO-1, NQO1, GCLM, GCLC, TXNRD1, TNXIP, FTH1, and GPX4 (Fig. 1J). Next, we further analyzed the dynamic changes in Nrf2 protein levels in PAMs post-PRRSV infection. When PAMs were infected with PRRSV at MOI of 0.01, 0.1, and 1, a dose-dependent decline in intracellular Nrf2 and HO-1 protein levels was observed with increasing PRRSV inoculum (Fig. 1K). Western blot analysis revealed a significant decrease in Nrf2 and HO-1 protein levels at 24, 36, and 48 hpi due to PRRSV infection (Fig. 1L). These results suggest that increasing PRRSV MOI decreases Nrf2 and HO-1 protein levels in a time- and dose-dependent manner in PAMs. Finally, to ascertain the necessity of PRRSV replication for decreasing Nrf2 levels, PRRSV was inactivated using ultraviolet (UV) light before inoculating PAMs. The results showed that only live PRRSV virions caused Nrf2 level reduction, while UV-inactivated PRRSV did not affect Nrf2 (Fig. 1M), indicating the requirement of PRRSV replication for inducing the decrease in Nrf2. As a crucial nuclear transcription factor, Nrf2 is activated in the cytoplasm upon oxidative and/or electrophilic stimuli and subsequently translocated into the nucleus (38). As shown in Fig. 1N, PRRSV infection decreased nuclear Nrf2 levels, impeding Nrf2 activation. These results imply that PRRSV weakens the Nrf2 antioxidant pathway, leading to cellular oxidative stress.

### Nrf2 inhibits PRRSV infection

**Fig 2:**
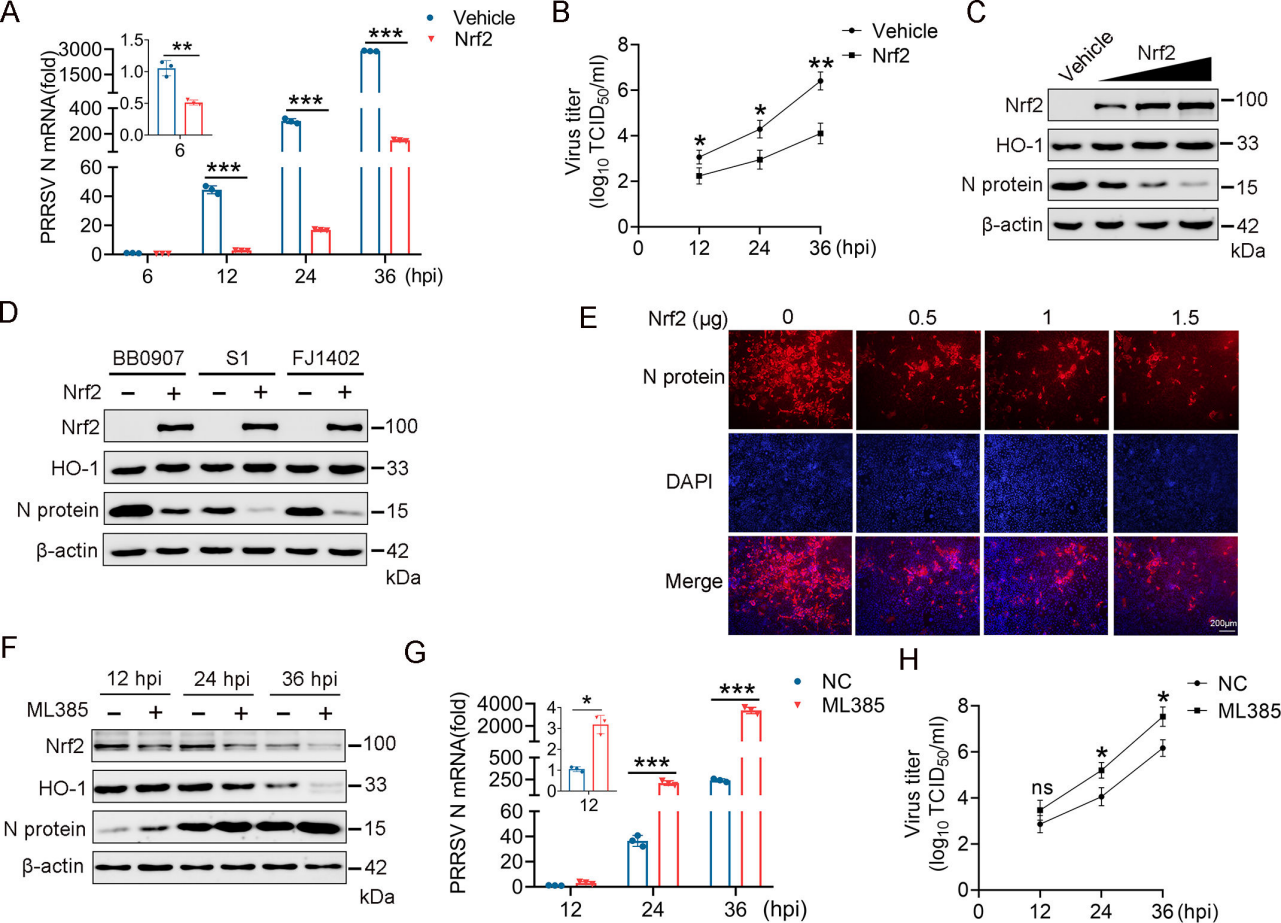
Nrf2 impairs PRRSV replication. (**A**) Marc-145 cells were transfected with either an empty vector or Nrf2 plasmid for 24 h and then infected with PRRSV (MOI = 0.1). Cells were collected at 6, 12, 24, and 36 hpi, and PRRSV N transcript levels were measured by RT-qPCR. (**B**) Cell supernatants were collected at different times post-infection (12, 24, and 36 hpi) to measure TCID_50_. (**C**) Marc-145 cells were transfected with control plasmid or different concentrations of Nrf2 plasmid (0, 0.5, 1, and 1.5 µg) for 24 h and then infected with PRRSV (MOI = 0.1). Protein expression was measured by western blotting. (**D**) Marc-145 cells were transfected with either the Nrf2 expression plasmid or an empty vector for 24 h and then infected with PRRSV strains BB0907, S1, and FJ1402 (MOI = 0.1) for 24 h. (**E**) Immunofluorescence analysis is shown to detect the PRRSV N expression (red) in Marc-145 cells and nuclei counterstained with DAPI in blue. (**F and G**) Inhibition of Nrf2 promotes PRRSV infection. PAM cells were treated with 2 µM of ML385 for 24 h and then infected with PRRSV for 12, 24, and 36 hpi. RT-qPCR and western blotting measured PRRSV N protein and Nrf2 mRNA levels. (**H**) Cell supernatants were collected at different times post-infection (12, 24, and 36 hpi) to measure TCID_50_. Data are expressed as means ± SD of three independent experiments (***P < 0.001, **P < 0.01, and *P < 0.05).

To explore the role of Nrf2 in PRRSV infection, Marc-145 cells were transfected with either an empty vector or a plasmid expressing Nrf2, followed by PRRSV infection 24 h later. As shown in Fig. 2A, the mRNA levels of PRRSV N were significantly reduced in Nrf2-overexpressing cells at 6, 12, 24, and 36 hpi. Additionally, the viral titers in the culture supernatants were lower in Nrf2-overexpressing cells compared with those of the control group (Fig. 2B). Subsequently, the impact of Nrf2 on PRRSV at the protein level was examined. Marc-145 cells were transfected with different doses of the Nrf2 plasmid and subsequently infected with PRRSV. Nrf2 overexpression increased the expression of HO-1 and significantly inhibited PRRSV replication, as evidenced by a dose-dependent reduction in PRRSV N protein expression (Fig. 2C). Moreover, Marc-145 cells overexpressing Nrf2 were infected with various PRRSV strains, including the highly pathogenic strain BB0907, classical strain S1, and NADC30-like strain FJ1402. The results indicated that Nrf2 overexpression inhibited all tested PRRSV strains (Fig. 2D). Immunofluorescence analysis also demonstrated that the fluorescence intensity of PRRSV N protein was significantly suppressed with increasing levels of Nrf2 expression in the cells (Fig. 2E). Our study confirms the inhibitory effect of Nrf2 on PRRSV infection.

Furthermore, we investigate the effect of Nrf2 inhibition on PRRSV infection using the Nrf2 inhibitor ML385. Marc-145 was treated with ML385 followed by PRRSV infection and showed increased mRNA and protein expression levels of the viral N protein over the 36 h infection period, indicating that PRRSV replication was enhanced when Nrf2 was silenced (Fig. 2F and G). Similarly, ML385 significantly increased the viral titers in the culture supernatants compared with the control group (Fig. 2H). These data imply that Nrf2 plays a regulatory role in controlling PRRSV replication. Overall, our findings demonstrate that Nrf2 overexpression inhibits PRRSV infection, while Nrf2 silencing enhances viral replication, highlighting the crucial role of Nrf2 in the host’s antiviral response against PRRSV.

### Nrf2 promotes IFN-β and ISG production following PRRSV infection

**Fig 3:**
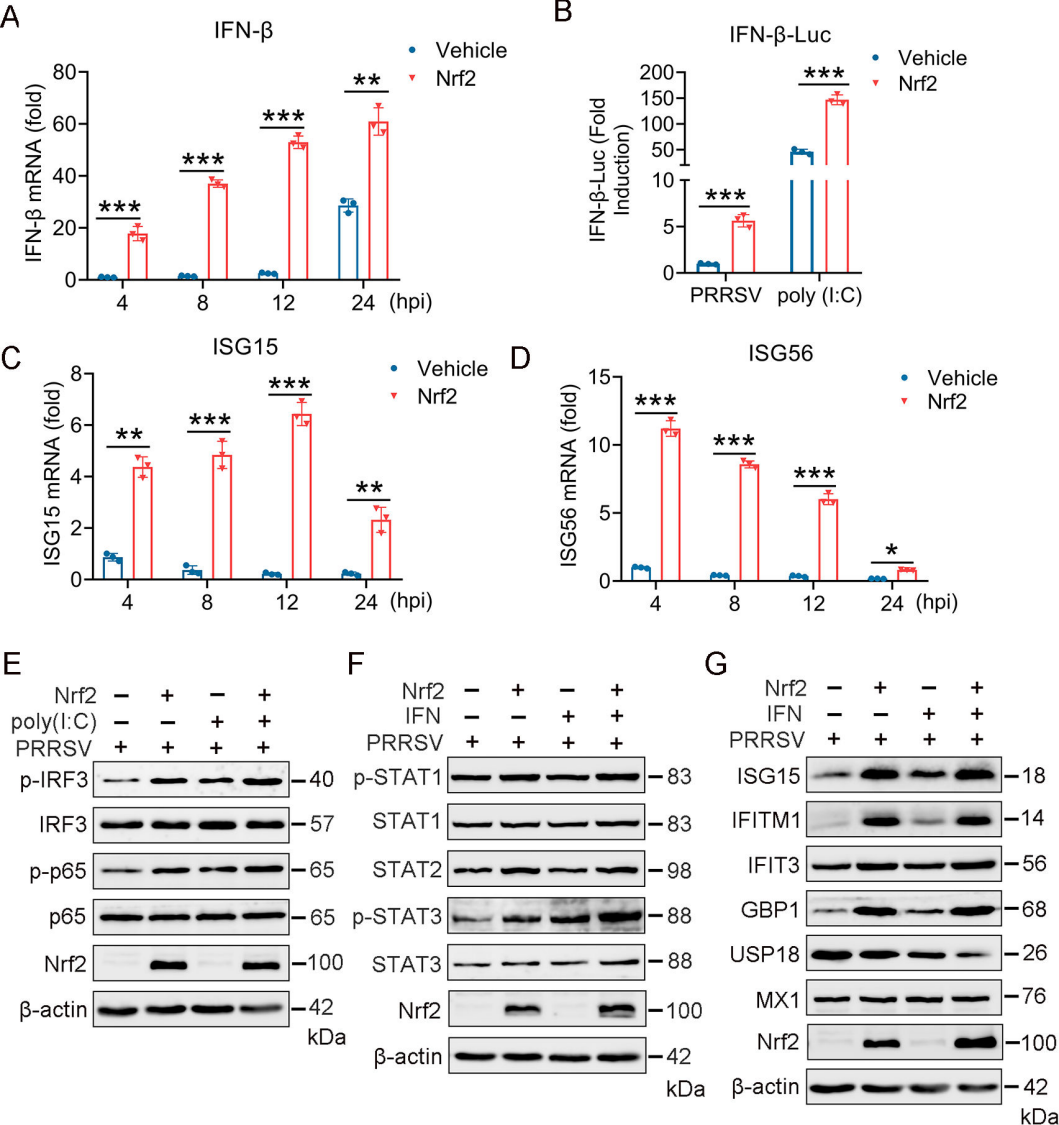
Nrf2 promotes IFN-β and ISG expression upon PRRSV infection. (**A**) Marc-145 cells were transfected with control or Nrf2 plasmids for 24 h and then infected with PRRSV for 4, 8, 12, and 24 h. Relative expression levels of IFN-β were measured by RT-qPCR, using GAPDH as an internal control. (**B**) Dual-luciferase assay measuring the effect of Nrf2 on IFN-β promoter activity upon PRRSV infection or poly (I: C) stimulation. (**C and D**) RT-qPCR analysis of the effect of Nrf2 on the expression levels of ISG15 and ISG56. (**E**) Western blot analysis of the effect of Nrf2 on IRF3 and p-IRF3 and p65 protein levels during PRRSV infection or poly (I: C) stimulation. (**F**) Effect of Nrf2 on STAT pathway activation during PRRSV infection or IFN stimulation. (**G**) Western blot analysis of the Nrf2’s effect on STAT and ISG protein expression during PRRSV infection or IFN stimulation. Data are expressed as means ± SD of three independent experiments (***P < 0.001, **P < 0.01, and *P < 0.05).

Previous studies have demonstrated that Nrf2 has an antiviral activity that functions mainly through the regulation of IFN-I production (39–41), and PRRSV is highly sensitive to IFN-I (42–46). Thus, the effect of Nrf2 on IFN-I production was assessed to preliminarily investigate the underlying anti-PRRSV mechanism of Nrf2. Marc-145 cells were transfected with a plasmid expressing Nrf2 and then infected with PRRSV for 4, 8, 12, and 24 h. Cells with activated Nrf2 expression exhibited a significant increase in IFN-β mRNA levels as early as 4 hpi and continued to show increased levels up to 24 h compared with cells transfected with an empty vector (Fig. 3A). To further investigate, we use a dual-luciferase reporter assay to assess the effect of Nrf2 on IFN-β promoter activity. The Nrf2 expression plasmid, IFN-β-Luc luciferase reporter plasmid, and pRL-TK were co-transfected into Marc-145 cells, followed by dual-luciferase activity analysis. The results showed that Nrf2 significantly activated the IFN-β promoter of PRRSV infection and poly (I: C)-induced cells (Fig. 3B). The induction of IFN-β requires the coordinated action of the transcription factors IRF3 and NF-κB. Western blot analysis revealed that Nrf2 significantly enhanced the levels of phosphorylated IRF3 (p-IRF3) and phosphorylated p65 (p-p65), which are indicators of IRF3 and NF-κB activation, respectively, following PRRSV infection and poly (I: C) induction (Fig. 3E). Altogether, these findings indicate that Nrf2 induces IFN-β production upon PRRSV infection.

IFNs are crucial in the host’s antiviral response by activating the JAK/STAT signaling pathway, leading to the expression of numerous ISGs (47, 48). Consistent with these findings, we investigated whether Nrf2 could regulate ISG expressions by modulating STAT signaling post-PRRSV infection. Marc-145 cells were transfected with Nrf2 expressing plasmid and then infected with PRRSV for 6 h and treated with IFN for 12 h. Western blot results showed that Nrf2 significantly upregulated the protein expression levels of phosphorylated STAT1 (p-STAT1), STAT2, and phosphorylated STAT3 (p-STAT3), following PRRSV infection and IFN induction (Fig. 3F). Additionally, Nrf2 increased the mRNA expression levels of ISG15 and ISG56 at all tested time points post-PRRSV infection (Fig. 3C and D). Moreover, Nrf2 upregulated the protein expression levels of ISG15, IFITM1, IFIT3, and GBP1 while downregulating the expression level of USP18 (Fig. 3G). Overall, these results suggest that the antiviral activity of Nrf2 against PRRSV is closely linked to the induction of IFN-β production and ISG expressions.

### PRRSV nsp5 reduces Nrf2 protein expression

**Fig 4:**
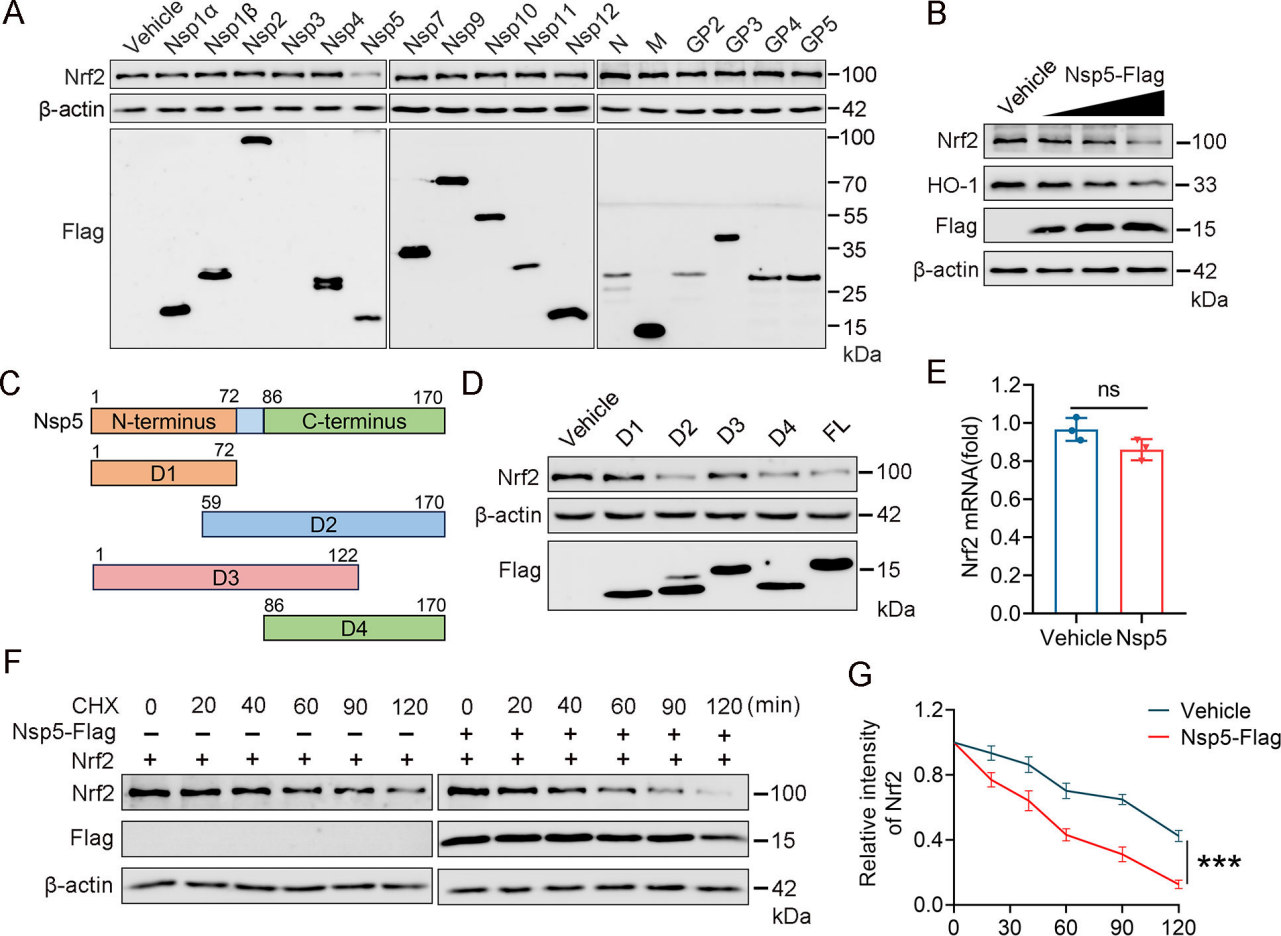
PRRSV nsp5 reduces Nrf2 protein expression. (**A**) HEK-293T cells were transfected with seventeen PRRSV proteins, and cell samples were collected at 48 h post-transfection. Western blot analysis was performed to assess the impact of PRRSV proteins on Nrf2 expression. (**B**) The effect of nsp5 doses (0 µg, 0.5 µg, 1 µg, and 2 µg) on Nrf2/HO-1 expression. (**C**) Schematic representation of the truncated mutants of PRRSV nsp5. Shown are four nsp5 mutants with amino acid residues: D1 (aa 1 to 72), D2 (aa 59 to 170), D3 (1 to 122), and D4 (86 to 170) C-terminal domain. (**D**) HEK-293T cells were transfected with four truncated mutants and nsp5 plasmids. Cells were harvested at 48 h post-transfection, and the impact on Nrf2 protein levels was assessed. (**E**) RT-qPCR analysis shows the effect of nsp5 on Nrf2 mRNA levels. (**F and G**) HEK-293T cells were transfected with Nrf2 alone or co-transfected with nsp5 for 28 h and then treated with cycloheximide for the indicated times. Lysates were analyzed by western blot. The relative intensity of Nrf2 was quantified using ImageJ software, and the average ± SD of three independent experiments is shown. ***P < 0.001, **P < 0.01, and *P < 0.05.

To identify which PRRSV-encoded protein regulates Nrf2 expression, we conducted an unbiased screening. HEK-293T cells were transfected with expression vectors for Flag-tagged PRRSV proteins. Cell samples were collected 48 h post-transfection, and Nrf2 expression was analyzed by western blotting. The result showed that PRRSV nsp5 significantly inhibited Nrf2 expression, while other viral proteins had minimal impact (Fig. 4A). To confirm this result, HEK-293T cells were transfected with different doses of the nsp5 plasmid. The results demonstrated that PRRSV nsp5 suppressed Nrf2 protein levels in a dose-dependent manner (Fig. 4B).

Additionally, to pinpoint the nsp5 domains responsible for Nrf2 degradation, we generated a series of truncated nsp5 mutants by analyzing the nsp5 polypeptide sequence using Lasergene (Fig. 4C). Four nsp5 truncated mutant fragments were cloned into the pCAGGS vector, and their overexpression was confirmed by immunoblotting using a Flag antibody. The results indicated that cells transfected with nsp5 D2 (amino acids [aa] 59 to 170) and nsp5 D4 (aa 86 to 170) had significantly lower levels of Nrf2 protein compared with those transfected with the empty vector. In contrast, nsp5 D1 (aa 1 to 72), corresponding to the N-terminal domain, had the least impact on Nrf2 levels, demonstrating that the C-terminal domain of nsp5 (aa 86 to 170) is responsible for inducing Nrf2 degradation (Fig. 4D).

The balance between protein synthesis and degradation determines intracellular protein levels (49), so we examined these factors both in cells with and without overexpression of nsp5. The results showed that nsp5 overexpression has no impact on Nrf2 mRNA levels (Fig. 4E) but did reduce Nrf2 protein levels, indicating that nsp5 influences Nrf2 stability. To test this hypothesis, we further co-transfected HEK-293T cells with Nrf2 and nsp5 and then treated the cells with cycloheximide, a protein synthesis inhibitor, before harvesting to analyze the degradation rate of Nrf2. As expected, nsp5 overexpression hastened the degradation of Nrf2 (Fig. 4F and G). Altogether, these data suggest that the C-terminal domain of nsp5 identified as aa 86 to 170 mainly induces Nrf2 degradation by disrupting its protein stability.

### Nsp5 interacts with p62 and inhibits p62-mediated Nrf2 signaling

**Fig 5:**
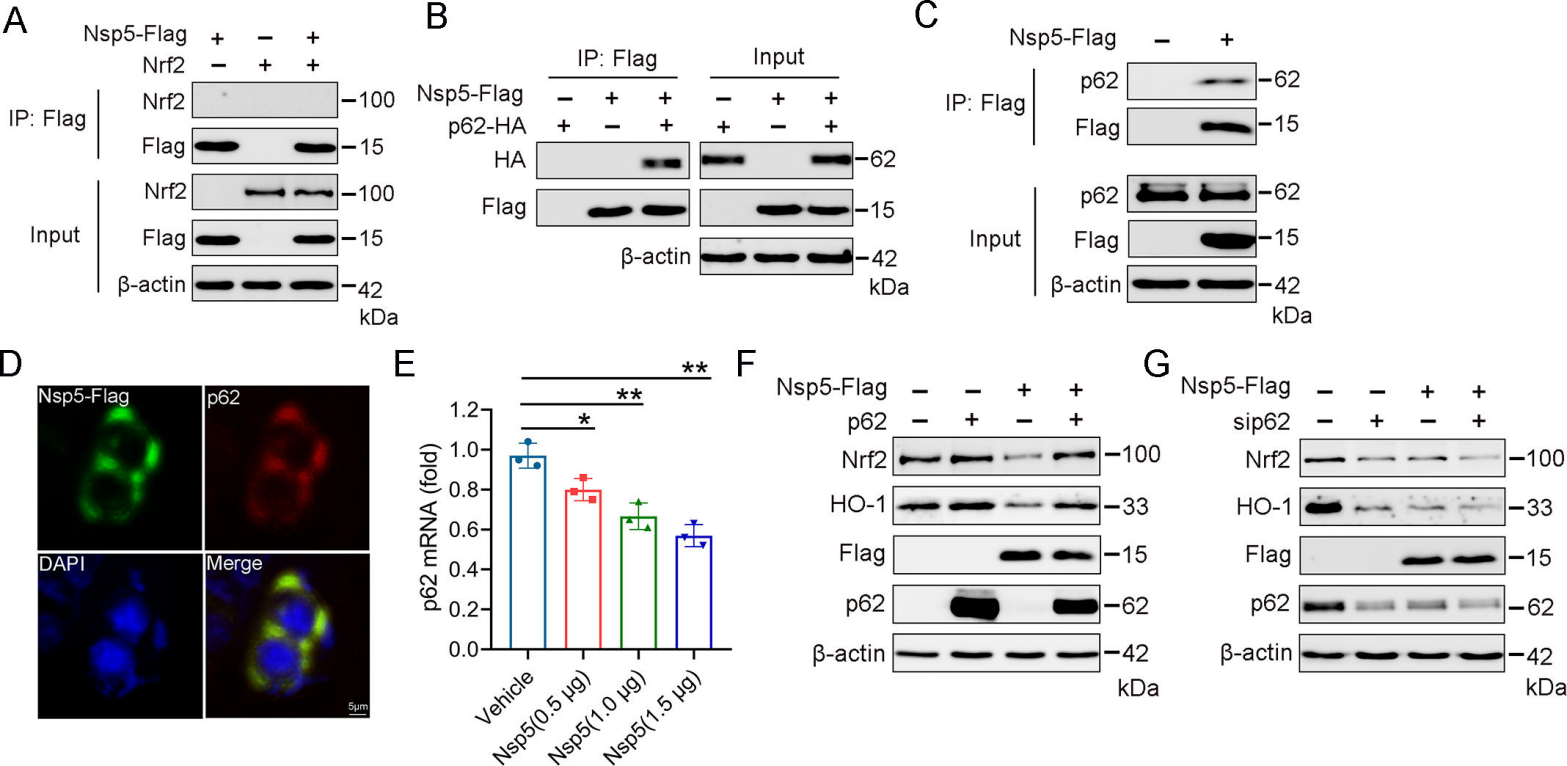
Nsp5 inhibits p62-mediated Nrf2 signaling. (**A**) HEK-293T cells were transfected with nsp5-Flag and Nrf2. After 36 h, cells were collected, and cell lysates were immunoprecipitated using anti-Flag magnetic beads. The precipitated proteins were detected with the indicated antibodies. (**B**) HEK-293T cells were transfected with nsp5-Flag and p62-HA for 36 h. Whole-cell lysates were immunoprecipitated with anti-Flag magnetic beads, and the precipitated proteins were detected with the indicated antibodies. (**C**) HEK-293T cells were transfected with nsp5-Flag for 36 h. Whole-cell lysates were immunoprecipitated with anti-Flag magnetic beads and analyzed by immunoblotting. (**D**) HEK-293T cells were transfected with Keap1-Myc and nsp5-Flag for 24 h, then fixed, stained, and observed using a confocal microscope. (**E**) The effect of nsp5 on p62 expression at the mRNA level. (**F**) HEK-293T cells were transfected with nsp5-Flag and p62-HA for 48 h, and the impact on Nrf2 was assessed. (**G**) HEK-293T cells were transfected with nsp5-Flag and sip62 for 48 h, and the impact on Nrf2 was assessed using western blot analysis.

To investigate how nsp5 inhibits Nrf2-mediated antioxidant pathways, we examined whether nsp5 directly interacts with Nrf2 to enhance its degradation. Co-immunoprecipitation assays showed no interaction between nsp5 and Nrf2 (Fig. 5A). Given that p62 is a necessary selective autophagy adaptor protein containing a Ub-associated domain that binds Keap1 (34), leading to Keap1 degradation and subsequent Nrf2 activation, we explored whether nsp5 can regulate Nrf2 through interaction with p62. HEK-293T cells were co-transfected with Flag-nsp5 and HA-p62, and co-immunoprecipitation analysis was performed. Cell lysates were precipitated using beads containing Flag-tag antibodies. The results showed that immunoprecipitation with an anti-Flag antibody pulled down both Flag-nsp5 and HA-p62, indicating an interaction between nsp5 and p62 (Fig. 5B). Additionally, we found that nsp5 also binds to endogenous p62 (Fig. 5C). Immunofluorescence assays further confirmed the co-localization of nsp5 with p62, supporting the co-immunoprecipitation results (Fig. 5D). To further investigate the role of p62 in nsp5-mediated Nrf2-related signaling, we observed that nsp5 inhibits the expression of p62 at the mRNA level in a dose-dependent manner (Fig. 5E). Overexpression of p62 increased the protein levels of Nrf2 and HO-1, thus activating the Nrf2/HO-1 antioxidant pathway. Co-transfection of nsp5 with p62 significantly reversed the nsp5-induced downregulation of Nrf2 and HO-1 expression (Fig. 5F). Conversely, silencing p62 with siRNA significantly reduced the expression of Nrf2 and HO-1 (Fig. 5J). These results demonstrate that nsp5 interacts with p62 and inhibits p62-mediated Nrf2 signaling. This suggests the evidence that nsp5 regulates the expression of Nrf2/HO-1 through p62.

### Effect of p62 on PRRSV replication

**Fig 6:**
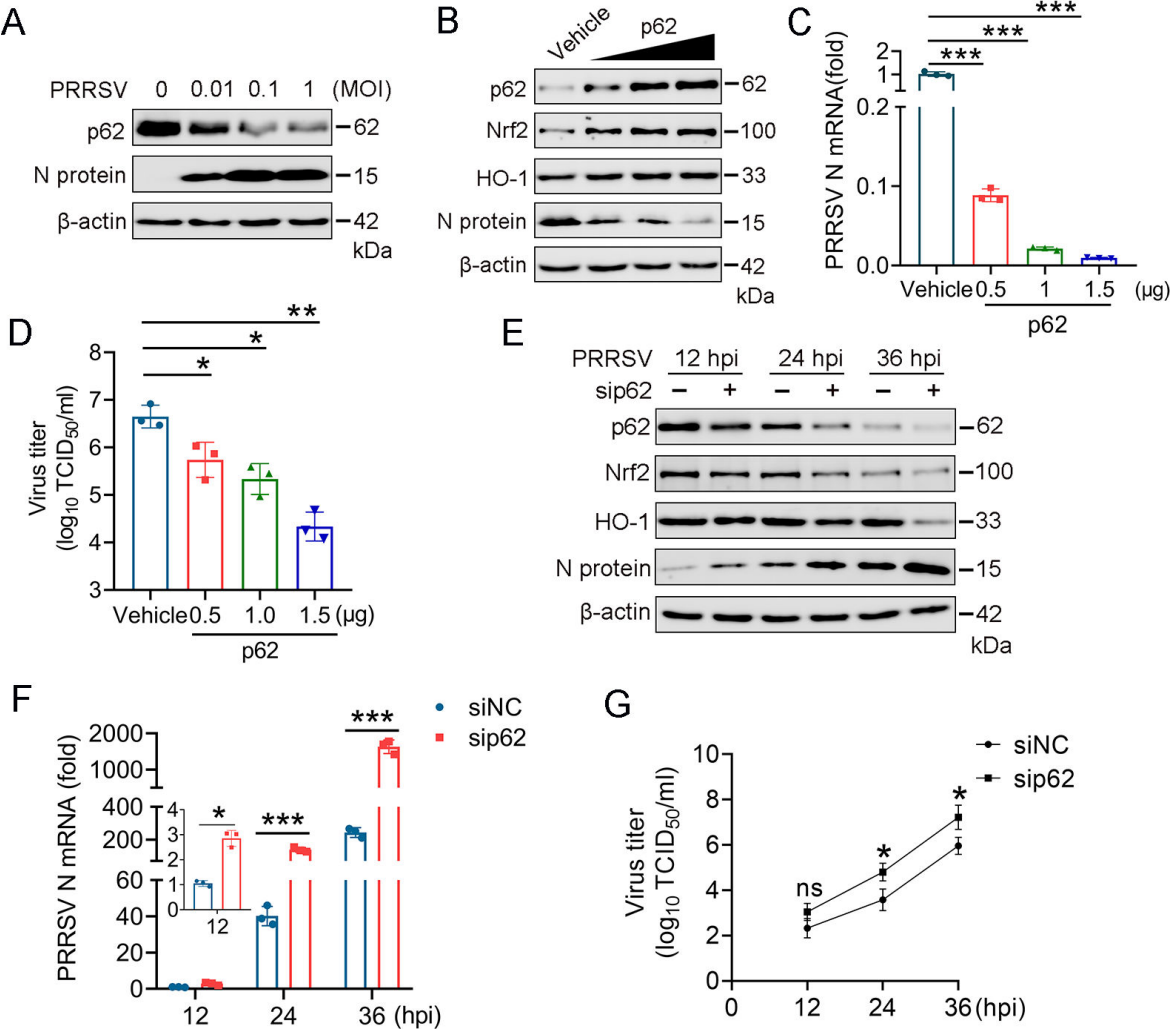
Effect of p62 on PRRSV replication. (**A**) PAMs were infected with PRRSV at MOI of 0.01, 0.1, and 1. After 48 h, the effect on p62 protein expression was assessed by western blotting. (**B and C**) Marc-145 cells were transfected with p62 plasmid for 24 h and then infected with PRRSV (MOI = 0.1). Cells were collected at 6, 12, 24, and 36 hpi. PRRSV N mRNA and protein levels were measured by RT-qPCR and western blotting. (**D**) Cell supernatants were collected at different times post-infection (12, 24, and 36 hpi) to measure TCID_50_. (**E and F**) Knockdown of p62 promotes PRRSV infection. Marc-145 cells were transfected with sip62 for 24 h and then infected with PRRSV. Cells were collected at 12, 24, and 36 hpi. PRRSV N and Nrf2 mRNA and protein levels were detected by RT-qPCR and western blotting. (**G**) Cell supernatants were collected at different times post-infection (12, 24, and 36 hpi) to measure TCID_50_. Data represent the results of three independent experiments (mean ± SD). Significant differences compared with the control group are denoted by ***P < 0.001, **P < 0.01, and *P < 0.05.

Since p62 positively regulates Nrf2-mediated signaling, we investigated the relationship between p62 and PRRSV replication. Western blot analysis was performed to assess p62 expression levels in PRRSV-infected PAMs. The results showed a dose-dependent decrease in p62 expression following PRRSV infection (Fig. 6A). To assess the impact of p62 on PRRSV replication, Marc-145 cells were transfected with either a p62 expression plasmid or an empty vector control, post-PRRSV infection. Viral replication levels over the 36 h infection period were analyzed using RT-qPCR and western blotting. The results indicated that overexpression of p62 significantly suppressed PRRSV N protein expression at both the mRNA and protein levels compared with the vector control (Fig. 6B and C). Additionally, the viral titers in the culture supernatants were reduced upon p62 overexpression (Fig. 6D). To further elucidate p62’s role, we also performed siRNA-mediated knockdown experiments to silence p62. The results demonstrated that p62 knockdown increased the mRNA and protein levels of the viral N protein and the viral titers in the supernatants of Marc-145 cells, compared with the control group (Fig. 6E through G).

### Nsp5 enhances Keap1-Nrf2 binding affinity by inhibiting p62, accelerating Keap1-mediated Nrf2 ubiquitination and degradation

**Fig 7:**
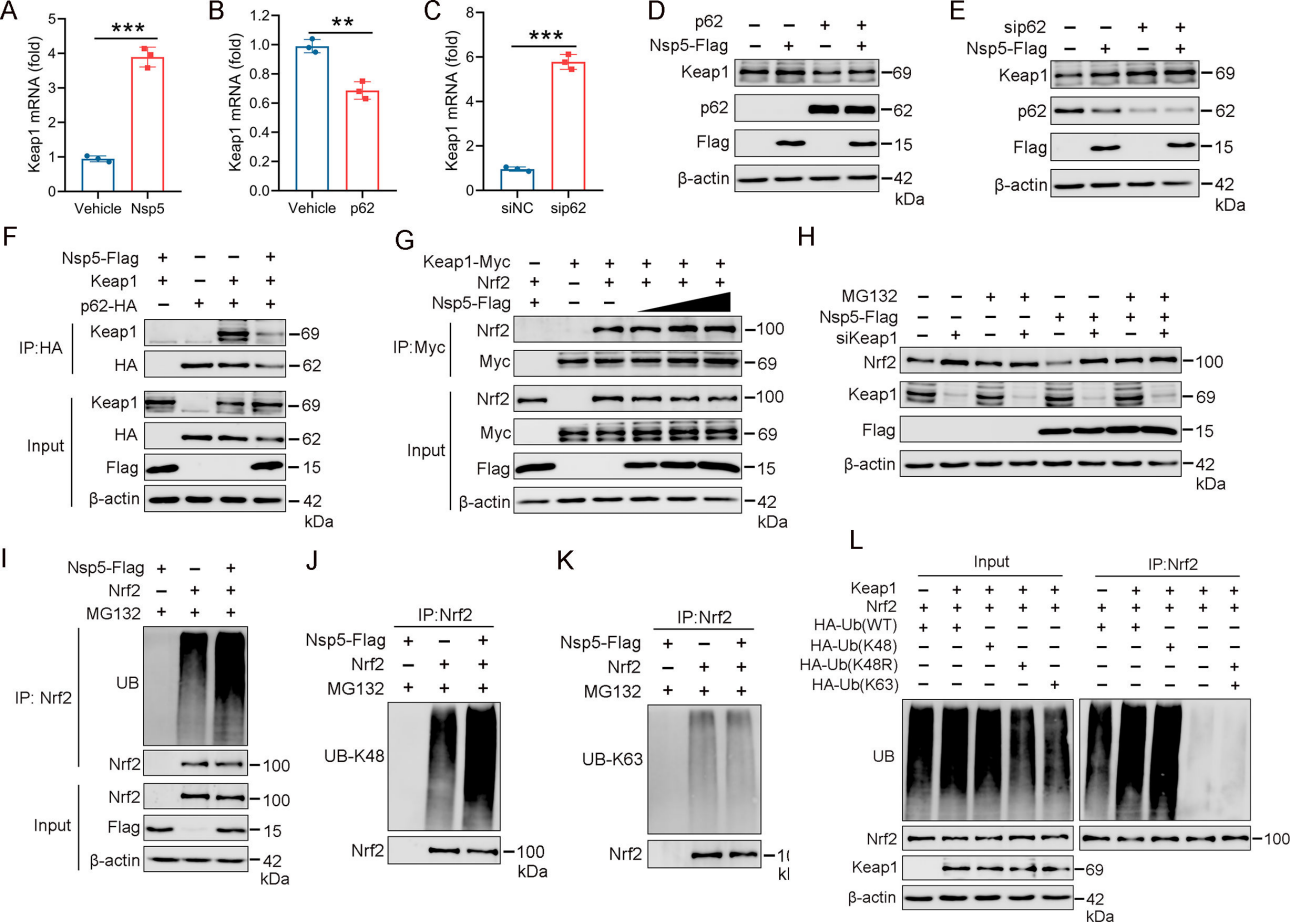
Nsp5 accelerates Keap1-dependent ubiquitination and degradation of Nrf2. (**A**) Effect of nsp5 on Keap1 mRNA levels. (**B and C**) Effect of p62 on Keap1 mRNA levels. (**D**) Effect of nsp5-Flag and p62 on Keap1 protein expression. (**E**) Effect of p62 knockdown on Keap1 protein expression. (**F**) HEK-293T cells were transfected with nsp5, p62-HA, and Keap1-Myc. Cells were collected 24 h post-transfection, and cell lysates were immunoprecipitated with anti-HA magnetic beads to assess the impact of nsp5 on the interaction between Keap1 and p62. (**G**) HEK-293T cells were transfected with Keap1-HA, Nrf2, and increasing doses of nsp5-Flag for 24 h, followed by treatment with MG132 (5 µM) for 2 h. Cells were harvested, and cell lysates were immunoprecipitated with HA magnetic beads. Immunoprecipitated proteins were analyzed by western blot with the Nrf2, Myc, and Flag antibodies. (**H**) HEK-293T cells were transfected with siRNA targeting Keap1 (si-Keap1), control siRNA (si-Ctrl), or nsp5 for 24 h. Total proteins were subjected to western blotting with the Nrf2, Keap1, and Flag antibodies in the presence or absence of MG132 (5 µM). (**I**) Effect of nsp5 on Nrf2 ubiquitination levels. HEK-293T cells were transfected with nsp5-Flag and Nrf2 for 24 h, followed by treatment with MG132 (5 µM) for 2 h. Whole-cell lysates were immunoprecipitated with anti-Nrf2 magnetic beads, and precipitated proteins were detected with the Ub, Nrf2, and Flag antibodies. (**J and K**) Analysis of Nrf2-bound precipitates using anti-UB-K48 and anti-UB-K63 antibodies. (**L**) HEK-293T cells were co-transfected with Keap1, Nrf2, and either HA-Ub_WT_, HA-Ub_K48_, HA-Ub_K48R_, or HA-Ub_K63_ antibodies for 24 h. Cell lysates were immunoprecipitated with an anti-Nrf2 antibody, and proteins were analyzed using a western blot with the specified antibodies. An empty vector was used to ensure equal amounts of plasmid DNA in each sample.

Keap1 serves as a substrate adaptor, bringing Nrf2 into the CUL3-dependent E3 Ub ligase complex, leading to rapid proteasome-mediated degradation of Nrf2 (50). To investigate whether Keap1 is involved in nsp5-induced Nrf2 degradation, we first examined the effects of nsp5 and p62 on Keap1 expression. Nsp5 increased Keap1 expression at the mRNA level (Fig. 7A). Conversely, p62 suppressed its expression at the mRNA level, while sip62 promoted it (Fig. 7B and C). Western blot results showed that nsp5 significantly increased Keap1 protein levels, while p62 inhibited Keap1 expression. Furthermore, p62 partially reversed the nsp5-induced increase in Keap1 expression (Fig. 7D; Fig. S1A). Similarly, p62 knockdown increased Keap1 expression (Fig. 7E; Fig. S1B). Co-immunoprecipitation experiments demonstrated that overexpression of nsp5 weakened the binding affinity between p62 and Keap1 (Fig. 7F). Additionally, nsp5 enhanced the interaction between Nrf2 and Keap1 in a dose-dependent manner (Fig. 7G). Taken together, these results suggest that nsp5 reduces the interaction between p62 and Keap1 while elevating Keap1 expression, thereby promoting the interaction between Keap1 and Nrf2.

To test whether nsp5-induced Nrf2 degradation relies on Keap1-mediated ubiquitination, we knocked down Keap1 using siRNA and assessed Nrf2 protein levels with or without MG132, a proteasome inhibitor. The result showed that Keap1 deficiency increased Nrf2 protein levels irrespective of nsp5 presence, and MG132 treatment restored the Nrf2 protein levels reduced by nsp5 (Fig. 7H). This suggests that nsp5 degrades Nrf2 through Keap1-mediated ubiquitination. To elucidate how nsp5 regulates Nrf2 through Keap1, we performed co-immunoprecipitation with anti-Nrf2 antibodies in the presence of MG132, followed by immunoblotting with anti-ubiquitin (Ub antibodies to detect ubiquitinated Nrf2. Interestingly, we observed that nsp5 enhanced Nrf2 ubiquitination (Fig. 7I). In addition, we determined that nsp5 promoted the formation of polyubiquitin chains linked to K48 on Nrf2 using anti-Ub-Lys48 (K48) and anti-Ub-Lys63 (K63) antibodies (Fig. 7J and K). To confirm this, we co-transfected Keap1 and Nrf2 along with HA-Ub (Ub_WT_), HA-Ub_K48_, HA-Ub_K48R_ (a K48 Ub mutant that causes premature termination of Ub chains), or HA-Ub_K63_. Our observations showed that overexpression of HA-Ub_K48_ effectively induced Nrf2 ubiquitination, while HA-Ub_K48R_ and HA-Ub_K63_ could not induce Nrf2 to produce K48 or K63 polyubiquitin chains (Fig. 7L). These results showed that nsp5 enhanced Keap1-mediated degradation of Nrf2 through K48-linked polyubiquitin.

### Nsp5 mutant of PRRSV plays a crucial role in the p62-mediated Nrf2 antioxidant pathway

**Fig 8:**
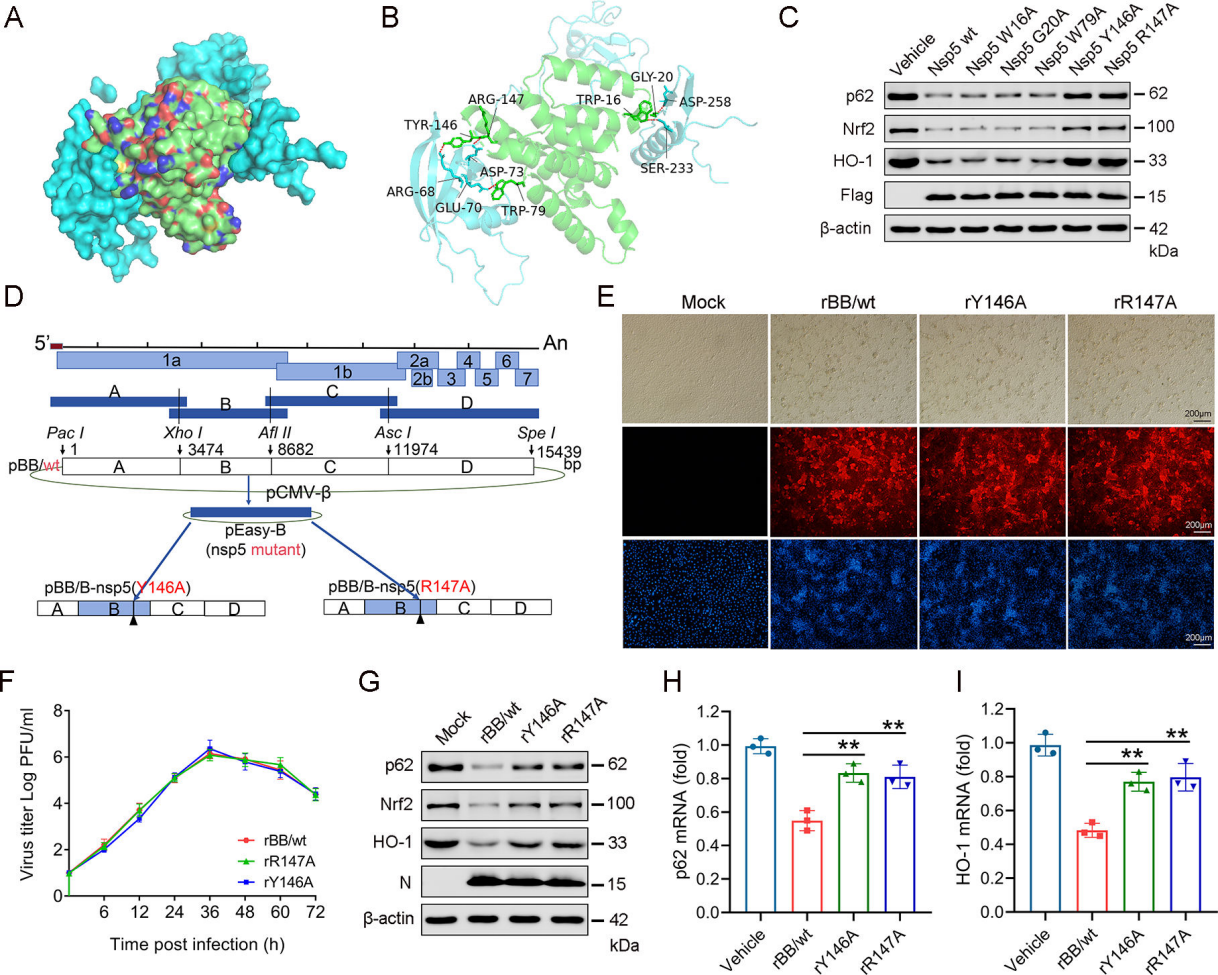
The nsp5 mutant virus plays a critical role in the p62-mediated Nrf2 antioxidant pathway. (**A**) Interaction analysis between nsp5 and p62 using the molecular docking server HDOCK. (**B**) Analysis of the interaction sites between nsp5 and p62 using the three-dimensional molecular structure visualization software Pymol. (**C**) Effects of nsp5 mutant plasmids on the expression of p62, Nrf2, and HO-1 proteins. (**D**) Schematic diagram of nsp5 mutant virus construction. (**E**) Full-length mutant cDNA clones pBB/Y146A and pBB/R147A were transfected into Marc-145 cells, and CPE light microscopy images, as well as immunofluorescence analysis of PRRSV N (red), were detected after 96 h. (**F**) Multi-step growth kinetics of PRRSV in Marc-145 cells after infection with the indicated virus strains (MOI: 0.1), with results expressed as TCID_50_. (**G**) Effects of nsp5 mutant viruses on the expression of p62, Nrf2, and HO-1 proteins. (**H**) Effects of nsp5 mutant viruses on p62 mRNA expression levels. (**I**) Effects of nsp5 mutant viruses on HO-1 mRNA expression levels.

To further elucidate the foundation of the interaction between nsp5 and p62, we utilized the HDOCK website and the software (Pymol 3) to conduct molecular docking and predict interaction sites between these two proteins. The molecular docking results revealed that nsp5 and p62 can bind through non-covalent interactions such as hydrophobic interactions and hydrogen bonds. As shown in Fig. 8A, where green represents the nsp5 protein and blue represents the p62 protein, the lowest binding energy score among the top 10 conformations of the molecular docking between nsp5 and p62 was −271.53. The most stable structure exhibited a binding energy of −395.67 Kcal/mol (Fig. S2). By selecting the most stable Model 1 for visual analysis, we found that W16, G20, W79, Y146, and R147 of nsp5 can form hydrogen bond interactions with S233, D258, E70, R68, and D73 of p62, respectively (Fig. 8B).

Based on the results of molecular docking predictions, mutations were introduced at the putative functional sites of nsp5 by replacing the target amino acid residues with alanine to obtain the W16A, G20A, W79A, Y146A, and R147A mutants. As shown in Fig. 8C, compared with nsp5 (wt), the Y146A and R147A mutants exhibited reduced inhibitory effects on the expression of p62, Nrf2, and HO-1 proteins. This suggests that amino acids at positions 146 and 147 of nsp5 are critical sites influencing the interaction with p62 and Nrf2.

To identify the key viral amino acids involved in suppressing the expression of the p62-mediated Nrf2 antioxidant pathway, we constructed mutant virus strains containing nsp5 mutations using an infectious cDNA clone of PRRSV (Fig. 8D). We successfully generated full-length mutant cDNA clones, pBB/Y146A and pBB/R147A, and transfected these plasmids into Marc-145 cells for 96 h to produce the mutant viruses rBB/wt, rY146A, and rR147A. These mutants induced cytopathic effects on Marc-145 cells and exhibited growth kinetics similar to those of rBB/wt (Fig. 8E and F). Subsequent analysis of Marc-145 cells infected with these mutants revealed that compared with rBB/wt, rY146A and rR147A had reduced inhibitory effects on the expression of p62, Nrf2, and HO-1 proteins, as demonstrated by western blot analysis. RT-qPCR analysis further showed that the mutants rY146A and rR147A enhanced the mRNA expression levels of p62 and HO-1. Altogether, these results showed that the residues Y146 and R147 of nsp5 play a crucial role in inhibiting the activation of the p62-mediated Nrf2 antioxidant pathway.

## DISCUSSION

The Nrf2/HO-1 pathway, an inducible antioxidant stress defense system, can be significantly upregulated under various stimuli, including hypoxia, cytokines, LPS, ROS, and infections (51, 52). ROS and free radicals act as cellular signaling molecules, influencing processes like the expression of proinflammatory mediators, including cytokines and chemokines (53). However, excessive ROS formation leads to oxidative stress, which is linked to the pathogenesis of several viruses, including influenza virus (54), Marburg virus (55), dengue virus (56), and hepatitis B virus (57), and in respiratory syncytial virus (RSV) (58). Consistent with these findings, our study demonstrated that PRRSV infection induces oxidative stress by increasing ROS and MDA production and downregulating antioxidant enzymes. Inhibition of Nrf2 is linked to a significant increase in virus entry and replication (59). We similarly demonstrated that PRRSV impairs the Nrf2 antioxidant pathway response. Additionally, to its role in oxidative stress response and heme metabolism, Nrf2/HO-1 pathway agonists inhibit a wide range of viral infections. Overexpressing Nrf2 has been shown to reduce the replication of IAV PR8 in alveolar epithelial cells (60). Nrf2 plays a critical role in preventing influenza virus-induced pulmonary inflammation and lung injury in mice (61). Similarly, our study provided evidence that overexpressing Nrf2 in Marc-145 cells inhibits PRRSV infection. Further studies supported the role of Nrf2 as a pivotal mechanism for protection against viral infection.

Type I IFNs (IFN-α/β) are crucial components of the innate antiviral immune response in virus-infected cells, preventing viral replication and limiting virus spread (62, 63). The Nrf2-targeting gene HO-1 plays a role in regulating innate immunity during viral infection (39), while Nrf2 itself can suppress IFN-β production (64, 65). Interestingly, Nrf2 disruption elevates IFN-β levels, suggesting that Nrf2 may preferentially inhibit the IFN-I system (65). However, the exact mechanism by which Nrf2 represses IFN-β expression upon PRRSV infection remains to be determined. In this study, we investigated whether Nrf2 can induce the IFN-β response during PRRSV infection to address this knowledge gap. The results showed that activated Nrf2 expression induced the production of IFN-β and ISGs. This suggests that the activation of Nrf2 leads to the upregulation of molecules that contribute to early pathogen recognition in the innate immune system, forming part of the antiviral response through the induction of IFN-β against PRRSV. Similar observations have been reported by Sun et al., who found that Nrf2 activation upregulates the expression of Toll-like receptors (TLR7), resulting in IFN-I induction (66). Increased IFN responses due to HO-1 activation by Nrf2 are part of the mechanisms that restrict Dengue virus infection in cell-based models and suckling mice (67). Importantly, natural Nrf2 inducers such as 2-hydroxymethyl-1,4-hydroquinone (SG-7) can enhance the IFN-I response by inducing Nrf2 expression (68). Accordingly, our study highlights the role of Nrf2 in inducing IFN-β production during PRRSV infection, suggesting that targeting the Nrf2/HO-1 axis could be a potential strategy for enhancing antiviral responses.

Like many viruses in the *Flaviviridae* family, PRRSV uses non-structural viral proteins to interfere with or degrade essential signaling components, thereby circumventing the IFN antiviral response (10, 69). For example, the non-structural DENV protein 2 protease complex promotes the degradation of Nrf2 through the lysosomal pathway (70). The SARS-CoV-2 nsp14 viral protein also inhibits Nrf2 by degrading sirtuin proteins 1 (15). Additionally, it has been reported that RING finger protein 4 mediates Nrf2 degradation in RSV (71). Consistent with these previous studies, we provided a novel function of PRRSV nsp5. Our study demonstrated that the C-terminal domain of nsp5, comprising amino acids 86 to 170, primarily induces Nrf2 degradation by disrupting its protein stability. This disruption of the Nrf2/HO-1-mediated antiviral response ultimately contributes to viral pathogenesis, with nsp5 playing a central role in viral-induced Nrf2 degradation. Our findings highlight the critical role of nsp5 in PRRSV’s strategy to evade the host’s antiviral defenses by targeting Nrf2, thereby promoting viral replication and pathogenesis.

Autophagy is a cellular defense mechanism against invading pathogens (72). As a key receptor protein, p62 recruits cargo to autophagosomes for lysosomal degradation (73). Recent studies have shown that SARS-CoV-2 main protease nsp5 targets p62 for cleavage at glutamic acid 354, thereby abolishing p62’s ability to mediate selective autophagy (74). Additionally, SARS-CoV-2 nsp13 induces selective autophagy degradation of TANK-binding kinase 1 by interacting with p62 (75). More recently, Wang et al. reported that nsp5 interacts with a member of the reticulophagy (ER-phagy) receptor family, specifically sequence similarity 134 member B (FAM134B), promoting its oligomerization and revealing a novel mechanism by which PRRSV utilizes FAM134B-mediated ER-phagy to evade host antiviral immunity (76). In the current study, PRRSV nsp5 interacts with p62 and inhibits p62-mediated Nrf2 signaling. This suggests that nsp5 disrupts the function of the selective autophagy receptor p62, preventing the degradation of the Nrf2 signaling components. While previous studies have identified pathways such as FAM134B (76) and SQSTM1 (74, 77) genes through which nsp5 of viruses such as PRRSV can degrade host factors, our current understanding mechanism focused on the nsp5 interaction with the regulation of p62-mediated Nrf2/HO-1 signaling. Overall, our study proved that nsp5 regulates the expression of Nrf2/HO-1 through p62 and Keap1 and suggests a potential viral strategy to counteract host immune defenses. Understanding this mechanism provides valuable insights into the development of targeted antiviral therapies.

Keap1, an adaptor for a cullin-3 (Cul3)-based Ub ligase, is a critical regulator of the cellular Nrf2 level (29). Our study demonstrated that nsp5 promotes Nrf2-Keap1 binding affinity by inhibiting p62, thereby reducing the p62-Keap1 interaction and increasing Keap1 expression (32). Competitively, p62 binds to Keap1, dissociating Nrf2 from Keap1 and forming the p62-Keap1-Nrf2 axis (33, 34). Importantly, the Nrf2-Keap1 relationship is disrupted by direct, high-affinity contact between the Marburg virus VP24 protein and the Kelch domain of Keap1, which leads to cytoprotection via transcriptional activation of the ARE promoter (78). In addition, the Keap1 Kelch domain interacts with p62, an autophagy factor that aids in the clearance of poly-ubiquitinated complexes, activating Nrf2 by disrupting its binding via Nrf2’s lower affinity Keap1 binding site (34, 79). According to recent reports, the NSs of the severe fever with thrombocytopenia syndrome virus can bind to the LC3 autophagy protein and so inhibit the production of autophagosomes. This inhibition may lead to the accumulation of p62 and suppression of p62-mediated autophagic degradation (80). Consistent with these findings, we demonstrated that nsp5 promotes Nrf2-Keap1 binding affinity by inhibiting p62, reducing the p62-Keap1 interaction, and elevating Keap1 expression. This process enhances Keap1-mediated ubiquitination and Nrf2 degradation through K48-linked polyubiquitin, suggesting a direct interaction between nsp5 and Keap1. Our data provided evidence that PRRSV has evolved mechanisms to engage the cellular Keap1 response as part of its replication strategy. Understanding these interactions offers valuable insights into developing targeted antiviral therapies aimed at disrupting the PRRSV nsp5-mediated manipulation of the Nrf2/Keap1 pathway.

Moreover, the docking of cyanines and squarylium dyes with SARS-CoV-2 proteases has demonstrated that these dyes could bind to nsp5 proteins in various conformations, indicating the potential for non-covalent binding between nsp5 and the dyes (81). More recently, it has been reported that there is a strong binding affinity (−30 Kcal/mol) of PRRSV nsp4 with three compounds, naringin dihydrochalcone, agathisflavone, and amentoflavone (82). Similarly, Pathak et al. reported the binding of PRRSV nsps, such as nsp4, to natural compounds sourced from ZINC (83). This cumulative evidence indicates that nsps exhibit a higher affinity for binding to molecules, suggesting that exploring these interactions could serve as viable approaches for designing inhibitors or treatments for viral infections in the development of novel antiviral agents. Consistent with this result, we conducted molecular docking analysis to deepen the understanding of the interaction between nsp5 and p62. The results reveal that nsp5 interacts with p62 through non-covalent interactions, particularly hydrogen bonds and hydrophobic interactions. Targeted mutations of predicted key amino acid residues confirmed that Y146 and R147 can partially reverse the inhibitory effect of nsp5 (wt) on p62, Nrf2, and HO-1 proteins. This suggests that Y146 and R147 are critical amino acid residues for nsp5 protein-induced oxidative stress in host cells. The construction of mutant virus strains and analysis in Marc-145 cells further corroborate the hypothesis that these amino acids are crucial in mediating viral suppression of the p62-mediated Nrf2 antioxidant pathway. These results provide valuable insights into the molecular dynamics of the nsp5-p62 interaction and highlight potential therapeutic interventions targeting the modulation of antioxidant defenses in host cells during PRRSV infection.

### Conclusion

**Fig 9:**
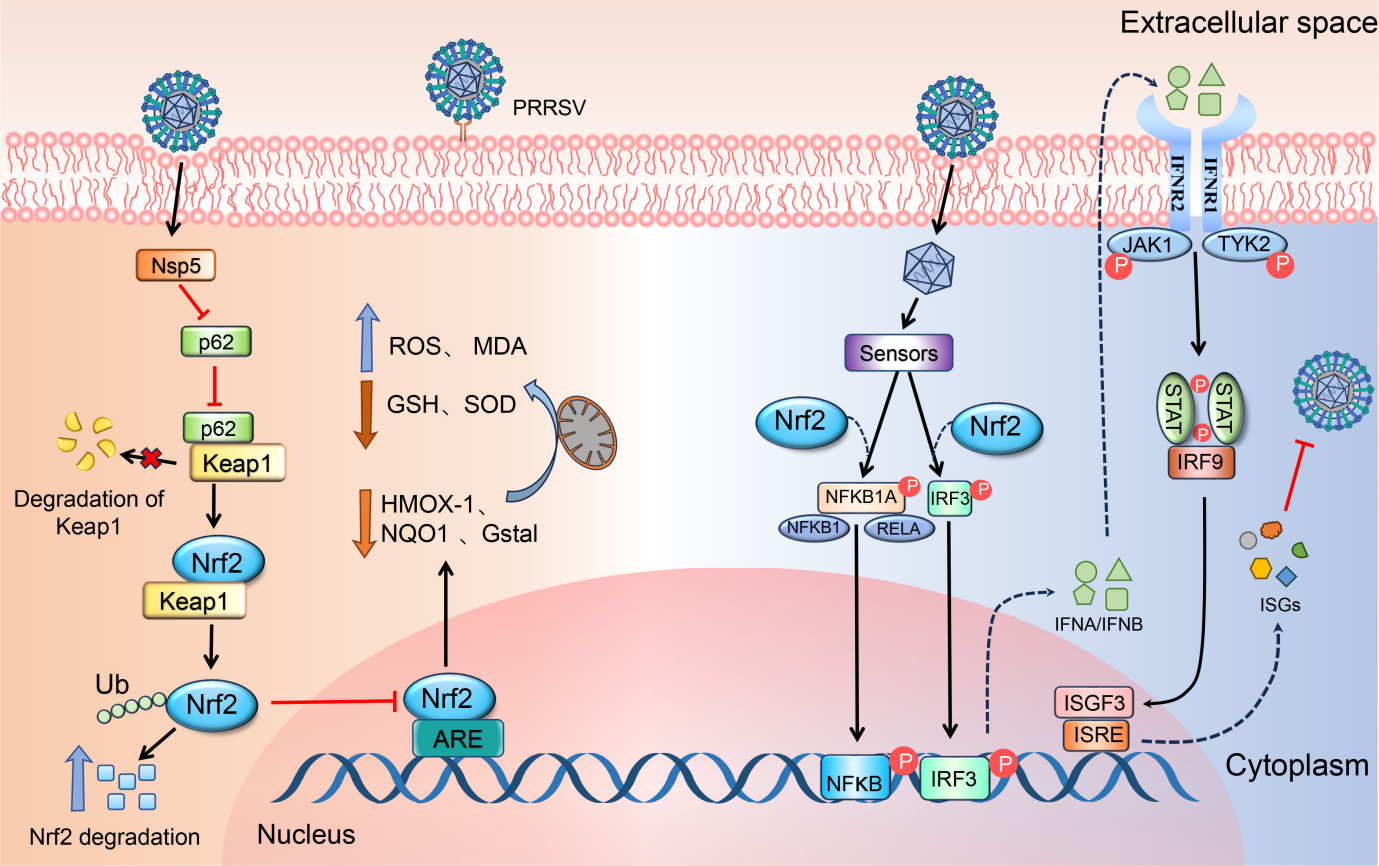
Schematic representation of PRRSV nsp5 protein interaction that accelerates the ubiquitination and degradation of Nrf2, thereby antagonizing its antiviral activity. PRRSV nsp5 aggravates host oxidative stress by inhibiting the activation of the p62/Keap1/Nrf2/HO-1 signaling pathway. The activation of Nrf2 induces the IFN-I signaling pathway and ISG expressions, which are crucial for inhibiting PRRSV proliferation.

Our study explores the interactions between PRRSV and the host p62/Keap1/Nrf2/HO-1 pathway, shedding light on the mechanisms behind nsp5-mediated PRRSV evasion strategies (Fig. 9). These insights enhance our understanding of PRRSV pathogenesis and suggest that targeting the p62/Keap1/Nrf2/HO-1 pathway could be a promising strategy for developing future PRRSV treatments.

## SUPPLEMENTAL MATERIAL

Supplemental material jvi.01585-24-s0001.docx Figures S1 and S2; Tables S1 to S3.

ASM does not own the copyrights to Supplemental Material that may be linked to, or accessed through, an article. The authors have granted ASM a non-exclusive, world-wide license to publish the Supplemental Material files. Please contact the corresponding author directly for reuse.
