# Supplementary material for: Porcine reproductive and respiratory syndrome virus nsp5 inhibits the activation of the Nrf2/HO-1 pathway by targeting p62 to antagonize its antiviral activity
Source: J Virol. 2025 Feb 28;99(4):e01585-24. doi: 10.1128/jvi.01585-24 (PMC11998497; doi:10.1128/jvi.01585-24)
Supplement: Supplemental material — Figures S1 and S2; Tables S1 to S3. [file jvi.01585-24-s0001.docx]

**Supplementary Materials:**

**
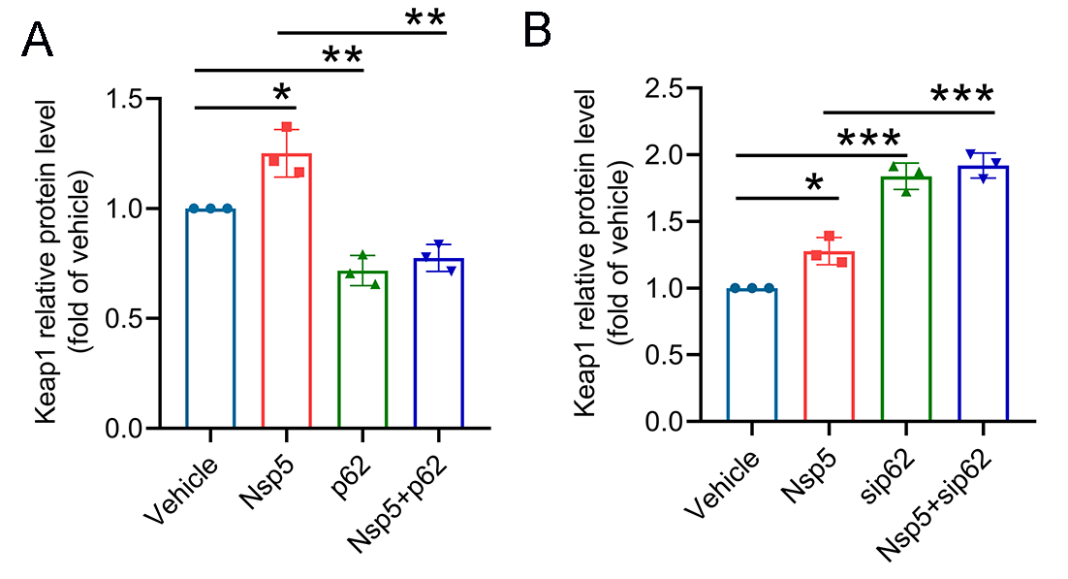
**

**Fig.S1.** Effects of Nsp5 and p62 on Keap1 protein expression. The relative intensity of Keap1 was quantified using ImageJ software, and the average ± SD of three independent experiments is shown. ***P < 0.001; **P < 0.01; *P < 0.05.

**
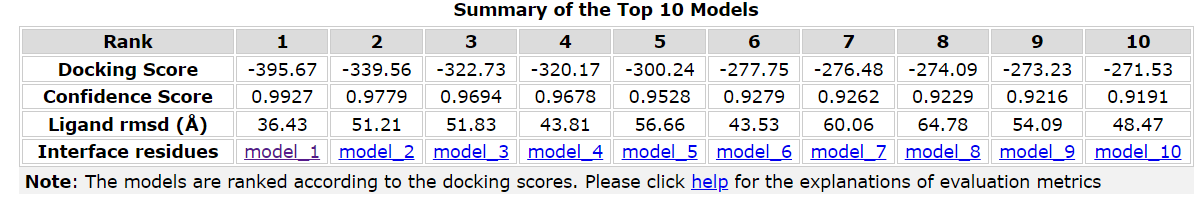
**

**Fig.S2.** A compilation of the binding energies for the first ten conformations resulting from the molecular docking of nsp5 and p62 has been conducted.

**Table S1.** Primers used for the construction of the nsp5 mutants

| Primer | Nucleotide Sequence (5'—3') |
| --- | --- |
| Nsp5-D1-F | CGGGGTACCATGGGAGGCCTTTCCACAGTTCAACTTC |
| Nsp5-D1-R | CCGCTCGAGTCACTTATCGTCGTCATCCTTGTAATCTGCTGTCAGAAGCCTGATCATCA |
| Nsp5-D2-F | CGGGGTACCATGCCATGGTCTGCGCAAGTCCTGATGA |
| Nsp5-D2-R | CCGCTCGAGTCACTTATCGTCGTCATCCTTGTAATCCTCGGCAAAGTATCGCAAGAAG |
| Nsp5-D3-F | CGGGGTACCATGGGAGGCCTTTCCACAGTTCAACTTC |
| Nsp5-D3-R | CCGCTCGAGTCACTTATCGTCGTCATCCTTGTAATCCATAACCATCATCCGGGGCAGG |
| Nsp5-D4-F | CGGGGTACCATGCTTGGTGCAGTAACCAGTTTTGTCG |
| Nsp5-D4-R | CCGCTCGAGTCACTTATCGTCGTCATCCTTGTAATCCTCGGCAAAGTATCGCAAGAAG |
| Nsp5-W16A-F | GTGTTTTTCCTCCTGGCGAGAATGATG |
| Nsp5-W16A-R | ATGCCCCATCATTCTCGCCAGGAGGAA |
| Nsp5-G20A-F | CTGTGGAGAATGATGGCGCATGCTTGG |
| Nsp5-G20A-R | GGGCGTCCAAGCATGCGCCATCATTCT |
| Nsp5-W79A-F | CTTAACAGAAATAGAGCGTCTCTTGGT |
| Nsp5-W79A-R | GTAAAAACCAAGAGACGCTCTATTTCT |
| Nsp5-Y146A-F | TTGTACTTGTTCAAGGCCCGCTGCCTC |
| Nsp5-Y146A-R | ATTGTGGAGGCAGCGGGCCTTGAACAA |
| Nsp5-R147A-F | TACTTGTTCAAGTACGCCTGCCTCCAC |
| Nsp5-R147A-R | GACATTGTGGAGGCAGGCGTACTTGAA |

F and R represent forward and reverse primers, respectively.

**Tab S2.** The sequences of siRNAs used in this study.

| Primer | Nucleotide Sequence (5'—3') |
| --- | --- |
| sip62 | CCAGACUACGACUUGUGUA |
| siKeap1 | GAGCGCCUCAUUGAAUUCGCCUACA |
| siNC | UUCUCCGAACGUGUCACGUTT |

**Tab. S3: The primer sequences for the relative real-time PCR assay.**

| Primer | Nucleotide Sequence (5’-3’) |
| --- | --- |
| mGAPDH-F | CCTTCCGTGTCCCTACTGCCAA |
| mGAPDH-R | GACGCCTGCTTCACCACCTTCT |
| mNrf2-F | ATTCAATGATTCTGACTCTG |
| mNrf2-R | CGTATCCCCAGAAGAATGTA |
| PRRSV-N-F | AAACCAGTCCAGAGGCAAG |
| PRRSV-N-R | TCAGTCGCAAGAGGGAAAT |
| mIFN-β-F | GCAATTGAATGGAAGGCTTGA |
| mIFN-β-R | CAGCGTCCTCCTTCTGGAACT |
| mISG15-F | CTGAAGGCAAAGATCGCCCA |
| mISG15-R | GTCGTTCCTCACCAGGATGC |
| mISG56-F | AGGAAACACCCACTTCGGTC |
| mISG56-R | CCTCTAGGCTGCCCTTTTGT |
| pGAPDH-F | GATCCCGCCAACATCAAAT |
| pGAPDH-R | TTCACGCCCATCACAAACAT |
| pNrf2-F | ATGCCCTCCTCTGCTACTT |
| pNrf2-R | CTTTCACGGTGGTCTTGGT |
| pHO-1-F | GGAGCACTCACAGCCCAACA |
| pHO-1-R | GTACAAGGACGCCATCACCAG |
| pNQO1-F | CCAAGTAGCCTCTTTGACCTA |
| pNQO1-R | ATGGATTTGCCCAAGTGAT |
| pGCLM-F | GGTTCATCTGTCCTTGGAGCAT |
| pGCLM-R | TTAAATCGGGCGGCATCAC |
| pGCLC-F | TCAGTAAGTCCCGATACGATT |
| pGCLC-R | TGATGAAGAGGTGAGCCAC |
| pTXNRD1-F | GGTGCTTGTGGTCTTTCCG |
| pTXNRD1-R | ACACGCTCATTGTCTTTGAT |
| pTNXIP-F | TCGGTCAGAGGCAATCACA |
| pTNXIP-R | CTTGGAGCCAGGGACACTA |
| pFTH1-F | GAACTTTGCCAAATACTTTCTTCAC |
| pFTH1-R | GCAGTCAGCCCATTCTCCC |
| pGPX4-F | GCTTTAGCCGCCTGTTCCG |
| pGPX4-R | CATGTGCCCGTCGATGTCC |

m means monkey, p means pig.
